# 16S rRNA gene-based microbiota profiles from diverse avian faeces are largely independent of DNA preservation and extraction method

**DOI:** 10.3389/fmicb.2023.1239167

**Published:** 2023-08-22

**Authors:** Johnson Edwards, Carmen Hoffbeck, Annie G. West, An Pas, Michael W. Taylor

**Affiliations:** ^1^School of Biological Sciences, University of Auckland, Auckland, New Zealand; ^2^New Zealand Centre for Conservation Medicine, Auckland Zoo, Auckland, New Zealand

**Keywords:** bird microbiota, avian, nucleic acids, DNA preservation, DNA extraction

## Abstract

The avian gut microbiota has been the subject of considerable recent attention, with potential implications for diverse fields such as the poultry industry, microbial ecology, and conservation. Faecal microbiotas are frequently used as a non-invasive proxy for the gut microbiota, however the extraction of high-quality microbial DNA from avian faeces has often proven challenging. Here we aimed to evaluate the performance of two DNA preservation methods (95% ethanol and RNAlater) and five extraction approaches (IndiSpin Pathogen Kit, QIAamp PowerFecal Pro DNA Kit, MicroGEM PrepGEM Bacteria Kit, ZymoBIOMICS DNA Miniprep Kit, and an in-house phase separation-based method) for studying the avian gut microbiota. Systematic testing of the efficacy of these approaches on faecal samples from an initial three avian species (chicken, ostrich, and the flightless parrot kākāpō) revealed substantial differences in the quality, quantity and integrity of extracted DNA, but negligible influence of applied method on 16S rRNA gene-based microbiota profiles. Subsequent testing with a selected combination of preservation and extraction method on 10 further phylogenetically and ecologically diverse avian species reiterated the efficacy of the chosen approach, with bacterial community structure clustering strongly by technical replicates for a given avian species. Our finding that marked differences in extraction efficacy do not appear to influence 16S rRNA gene-based bacterial community profiles provides an important foundation for ongoing research on the avian gut microbiota.

## 1. Introduction

A role for the gut microbiota in vertebrate host health is by now well established, with implications for host digestion, pathogen defence, reproduction, and even behaviour (Gilbert et al., 2018; NIH Human Microbiome Portfolio Analysis Team, 2019). With cultivation-based approaches providing an incomplete picture of microbial diversity for most environments, including the gut, the microbiota is typically assessed via the extraction and subsequent analysis of microbial DNA (Hugenholtz et al., 1998; Talaro et al., 2013; Grond et al., 2018). The faecal microbiota is widely used as a standard, non-invasive proxy for bacterial communities in the gut, with faeces representing an ideal sampling source when describing the microbiota of threatened species for which destructive sampling is not an option. The DNA obtained should ideally be of both high quality and high quantity to facilitate downstream processes such as PCR amplification and DNA sequencing. DNA extraction can be achieved via a range of approaches which are known to represent a significant source of technical variation that influences microbial community profiles (Costea et al., 2017; Douglas et al., 2020).

Despite the success of cultivation-independent methods in obtaining and analysing high-quality DNA from mammalian (including human) faecal samples, these have not always achieved the same level of success when applied to avian faeces (Jedlicka et al., 2013; Vo and Jedlicka, 2014; Eriksson et al., 2017). This may be attributable, at least in part, to specific aspects of the avian anatomy, such as the existence of a cloaca. Here, faecal matter is mixed with contents of the urogenital and reproductive systems, producing a faecal matrix that is physico-chemically complex and sometimes problematic for the extraction of bacterial DNA of sufficient quantity and quality. Our previous works on avian microbiotas (Waite and Taylor, 2014; Perry et al., 2017; West et al., 2022) have identified considerable variation in DNA extraction efficacy amongst avian species, different extraction methods and even amongst individuals of the same species. A recent study by Eriksson and colleagues (Eriksson et al., 2017) evaluated several methods for extracting microbial DNA from avian faeces, but focused primarily on *Campylobacter* rather than the overall microbiota. Another study compared three DNA extraction methods across three bird species representing distinct dietary guilds, namely granivore, omnivore, and carnivore (Hou et al., 2021). The optimal approach in that study varied depending on avian species and the criterion used to assess efficacy (e.g., alpha-diversity, cell lysis capacity). Five commonly used DNA preservation methods were compared by Vargas-Pellicer and colleagues for their ability to maintain consistent microbial taxonomic ratios in faecal samples obtained from a passerine (Vargas-Pellicer et al., 2019). That study showed that choice of preservation method significantly influenced the bacterial community profile recovered, particularly regarding the ratios between dominant phyla such as *Firmicutes* and *Proteobacteria*, but there was no significant impact on the quantity or quality of nucleic acids obtained. To date, a standardised protocol for both the preservation and extraction of high-quality microbial DNA from avian faeces has not been established but would be beneficial for the field, particularly given the recent surge of interest in avian gut microbiology (Bodawatta et al., 2022).

In this study we took a two-pronged approach to identify and apply an effective methodology for preserving and extracting bacterial DNA from the faeces of diverse avian hosts. First, we undertook an in-depth analysis of faecal samples from three taxonomically and ecologically diverse avian species to determine a satisfactory combination of preservation solution and DNA extraction method. These analyses encompassed measures of DNA yield, quality and integrity, as well as the sequencing of 16S rRNA gene amplicons. Second, in order to determine its wider applicability, we applied our selected combination of methods to the 16S rRNA gene-based analysis of a further 10 host species from across the avian tree of life. Surprisingly, 16S rRNA gene profiles were largely the same in each avian species, regardless of preservation or extraction method used to derive bacterial DNA. This study should prove valuable for researchers investigating the avian gut microbiota in diverse contexts such as the poultry industry, threatened species conservation, and avian ecology.

## 2. Materials and methods

### 2.1. Study species

To test the efficacy of multiple preservation and DNA extraction methods, faecal samples were collected from three phylogenetically and ecologically distinct avian species in July 2021: chicken (*Gallus gallus domesticus*), ostrich (*Struthio camelus*), and kākāpō (*Strigops habroptilus*). Chicken faeces were collected from a domestic household, ostrich samples from Auckland Zoo, Auckland, New Zealand, and kākāpō samples from sick and recovering individuals at the New Zealand Centre for Conservation Medicine, Auckland Zoo. All samples were collected into sterile 50 mL polypropylene tubes as aseptically as possible. Once we identified a suitable combination of preservation and extraction methods for obtaining high quality and quantities of DNA from these three species, we collected faecal samples from an additional 10 avian species housed at Auckland Zoo: blue penguin (*Eudyptula minor*), brolga crane (*Gus rubicunda*), brown kiwi (*Apteryx mantelli*), flamingo (*Phoenicoparrus roseus*), kereru/wood pigeon (*Hemiphaga novaeseelandiae*), kookaburra (*Dacelo novaguineae*), rainbow lorikeet (*Trichoglossus moluccanus*), ruru/morepork (*Ninox novaeseelandiae*), whio/blue duck (*Hymenolamius malacorhynchos*), and zebra finch (*Taeniopygia guttata castanotis*). These latter samples were collected into sterile 50 mL polypropylene tubes by Auckland Zoo personnel from within the individual birds’ enclosures.

### 2.2. Sample collection and preservation

**Figure 1:**
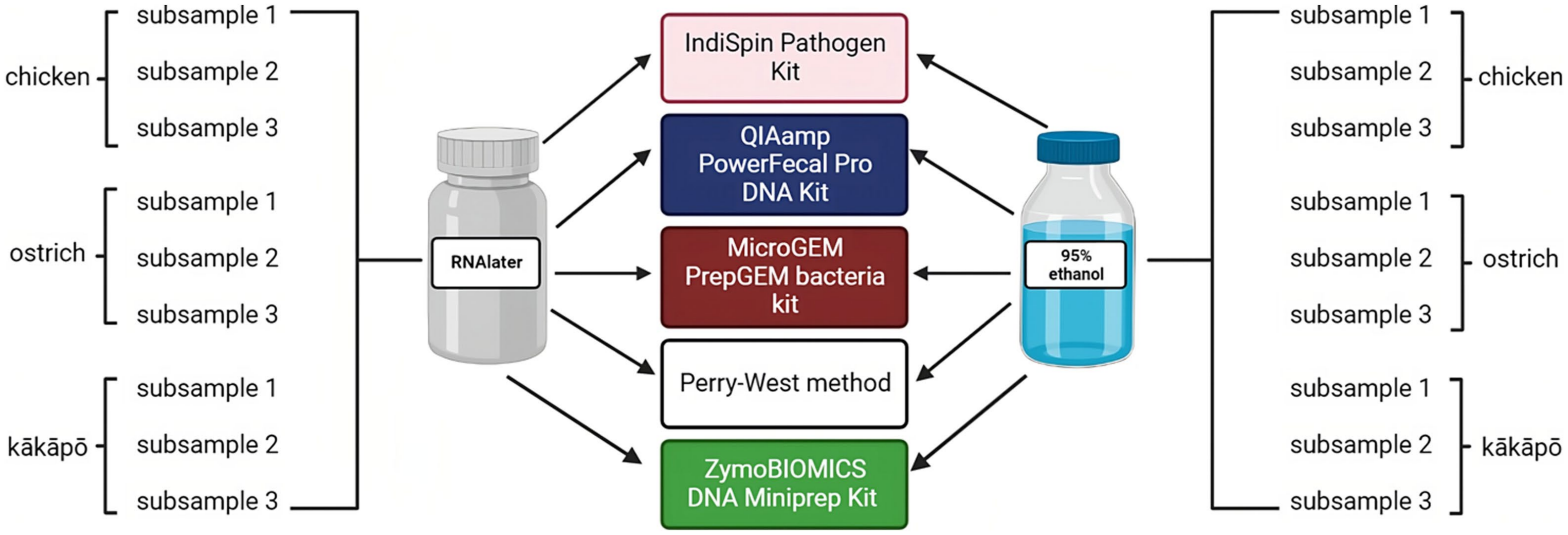
Sampling scheme to evaluate influence of preservation and DNA extraction methods on analysis of the avian microbiota. Two preservation methods (RNAlater and 95% ethanol) and five DNA extraction methods (Perry-West, QIAamp PowerFecal Pro DNA Kit, IndiSpin Pathogen kit, MicroGEM PrepGEM Bacteria Kit, and ZymoBIOMICS DNA Miniprep Kit) were applied to three subsamples of the pooled faeces from each of kākāpō, chicken, and ostrich. In total, 90 (sub)samples of avian faeces were analysed in this part of the study. Created with BioRender.com.

For each avian species, faecal samples were collected from multiple (≥3) individuals then pooled to reduce any effect of inter-individual variability. Pooled samples were homogenised using a sterile metal spatula. From each pooled sample, 600–1,000 mg of faeces were removed and placed in either 3 mL of 95% ethanol for storage at room temperature [following the findings of Marotz and co-workers (Marotz et al., 2021)], or 3 mL of RNAlater for incubation at 4°C overnight and subsequent storage at −20°C (Figure 1). RNAlater and ethanol (either 70% or 95% concentration) are both common preservation methods for field-collected samples from which DNA is to be extracted (Shokralla et al., 2010; Moreau et al., 2013). Though RNAlater is primarily marketed for its ability to stabilise RNA, it is also effective for DNA preservation. We selected these two preservation methods in order to compare two widely used and available chemicals and their downstream effects during extraction.

### 2.3. DNA extraction

Four commercial kits and one non-kit based method were chosen for evaluation of DNA extraction efficacy from kākāpō, chicken, and ostrich faecal samples: IndiSpin Pathogen Kit, QIAamp PowerFecal Pro DNA Kit, MicroGEM prepGEM Bacteria Kit, ZymoBIOMICS DNA Miniprep Kit, and the Perry-West method of extraction (Perry et al., 2017; West et al., 2022). The input quantity of faeces was based on the manufacturer’s recommendation for each kit; where a range of input sample quantity was given the maximum was chosen (Table 1). Elution volume was based on the recommended volume for each kit; where a range was given the midpoint was chosen. To remove RNAlater and ethanol from all samples, samples were centrifuged at 13,000× *g* for 1 min and supernatant removed. 1 mL of PBS was then added and the solution was vortexed for 30 s before centrifuging at 13,000× *g* for 5 min, followed by removal of the supernatant (Hou et al., 2021). Each sample was subsampled in triplicate and processed using each extraction approach (Table 1), along with an extraction blank containing no faecal material. Once an appropriate preservation and extraction method were selected following quality and quantity assessment and 16S rRNA gene amplicon sequencing, this combination of methods was applied to a further 10 avian species in order to evaluate its broader applicability.

**Table 1:** Comparison of the five microbial DNA extraction approaches used in this study.

Extraction approach (abbreviation)	Sample input quantity (mg)	Lysis type	Elution volume (μL)	DNA isolation method	Reference (if applicable)
IndiSpin Pathogen Kit (I)	150	Mechanical, enzymatic	100	Spin column	Eriksson et al. (2017)
QIAamp PowerFecal Pro DNA Kit (PF)	250	Mechanical, chemical	75	Spin column	
MicroGEM prepGEM Bacteria Kit (PB)	50	Enzymatic, thermal	100	Phase separation	
Perry-West method (PW)	200	Mechanical	20	Phase separation	Perry et al. (2017), West et al. (2022)
ZymoBIOMICS DNA Miniprep Kit (Z)	200	Mechanical, chemical	100	Spin column	

Unless otherwise stated, commercial kits were utilised according to the manufacturer’s recommendations. For the IndiSpin kit, the protocol was modified in line with the findings of Eriksson and co-workers (Eriksson et al., 2017).

DNA quantity was measured using a Qubit® 3.0 Fluorometer and DNA quality evaluated on an Implem Nanophotometer N60 spectrophotometer to obtain the A260/A280 nm ratio. Quality ratios of 1.8–2.0 were considered to be pure DNA (Bunu et al., 2020). DNA extracts were visualised on 1% agarose gels to examine DNA shearing and integrity.

### 2.4. 16S rRNA gene amplification and sequencing

The V3–V4 region of the 16S rRNA gene was amplified from extracted DNA using the 341F-806R primer pair (Klindworth et al., 2013). PCR thermal cycling conditions involved initial denaturation at 95°C for 3 min, 35 cycles of denaturation at 95°C for 20 s, annealing at 57°C for 15 s, and extension at 72°C for 30 s, then a final elongation step at 72°C for 1 min (West et al., 2022). PCR products were also visualised on 1% agarose gels to ensure the presence of amplified DNA. Amplified 16S rRNA genes were then purified using a ZR-96 DNA Clean-up Kit (Zymo Research, Seattle, USA) before sequencing by Auckland Genomics Ltd. using Illumina MiSeq (2 × 300 bp chemistry). Raw 16S rRNA gene sequences generated in this study were uploaded to the Sequence Read Archive (BioProject ID: PRJNA981578).

### 2.5. Bioinformatic analysis

16S rRNA gene amplicon sequences were processed using the DADA2 package in R (version 4.0.1). Primer regions were removed using Trimmomatic (Bolger et al., 2014) and reads were trimmed to 280 bp for forward reads and 240 bp for reverse reads following quality assessment, followed by merging of forward and reverse reads. Taxonomy was assigned using the SILVA 138 ribosomal RNA database (Quast et al., 2012; Callahan et al., 2016). Sequence chimaeras were removed, and the remaining reads were analysed using the phyloseq package in R (ver 1.42.0) (McMurdie and Holmes, 2013). Before analysis, all samples were compared with sequenced extraction blanks and contaminants were removed using the R package decontam (ver 1.18.0) (Davis et al., 2018). All figures were produced using ggplot2 in R (ver 3.4.0) (Wickham, 2011).

Alpha-diversity was calculated from rarefied data using the GUniFrac package in R (ver 1.7). Sequences from the initial (kākāpō, chicken, ostrich) comparisons were rarefied to 13,000 reads/sample, whilst those involving application of the chosen combination of methods to 10 further avian species were rarefied to 2,000 reads/sample. Beta-diversity was assessed using the Bray–Curtis dissimilarity metric and visualised by non-metric multidimensional scaling (nMDS). The effect of preservation method, DNA extraction method, and avian species on 16S rRNA gene-based bacterial community profiles was calculated using PERMANOVA in the adonis2 function of the R Vegan package (ver 2.6–4) (Dixon, 2003).

## 3. Results

### 3.1. Determining the influence of preservation and extraction method on DNA recovered from avian faeces

#### 3.1.1. DNA quality, quantity and integrity

**Figure 2:**
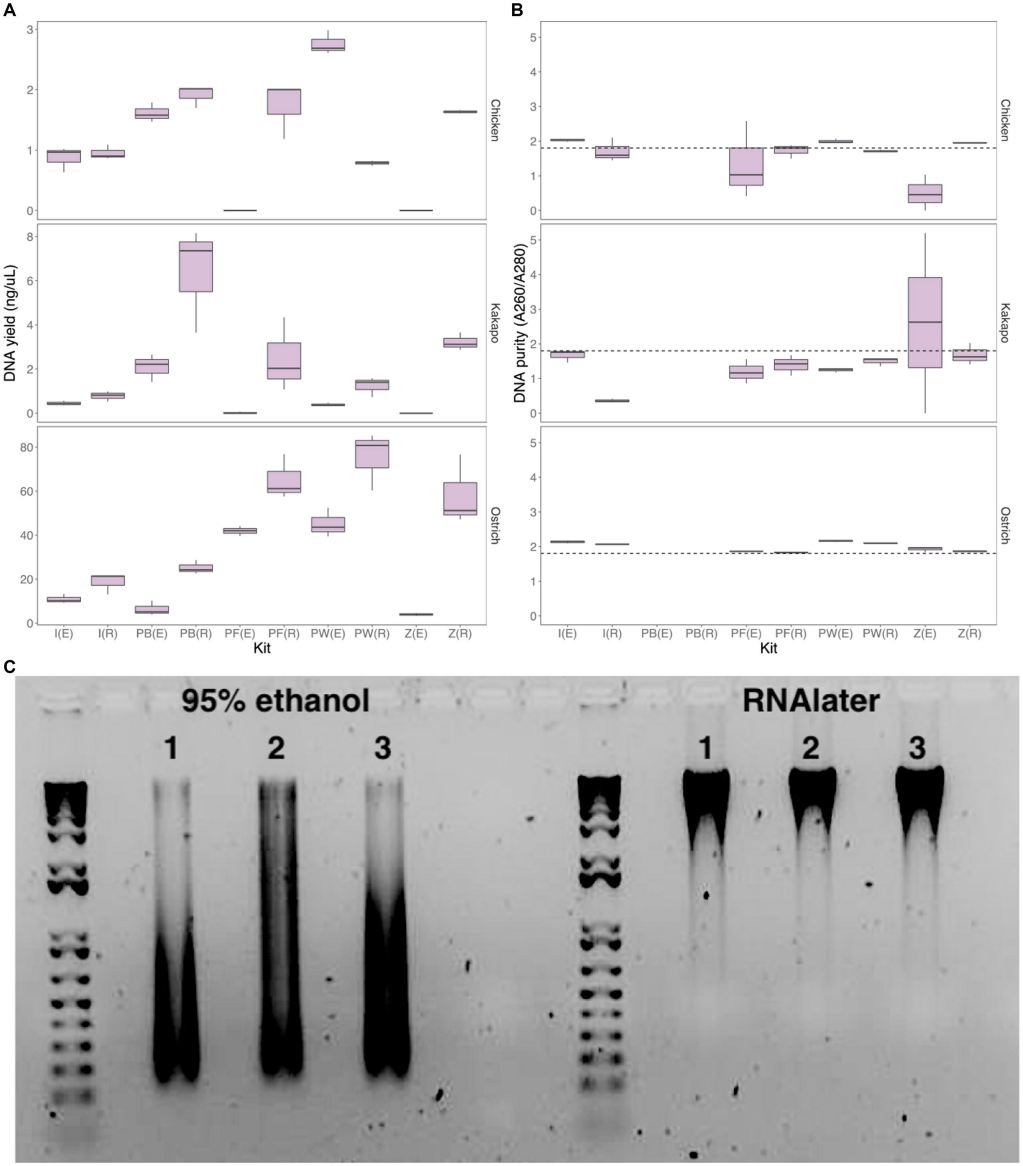
Yield, purity and integrity of extracted DNA. **(A)** DNA yield (ng/uL) and **(B)** DNA purity (A260/A280 nm ratio) for chicken (log-transformed), kākāpō, and ostrich faecal samples using two preservation methods and five extraction approaches (*n* = 3 technical replicates for each preservation/extraction method combination). The dotted line in panel **(B)** represents an A260/A280 nm ratio of 1.8, the desired quality for DNA in downstream analysis. [(E), 95% ethanol; (R), RNAlater; I, IndiSpin Pathogen Kit; PB, MicroGEM prepGEM Bacteria Kit; PF, QIAamp PowerFecal Pro DNA Kit; PW, Perry-West method; Z, ZymoBIOMICS DNA Miniprep kit]. **(C)** Representative agarose gel image of ostrich samples (3 technical replicates) preserved in 95% ethanol (left) and RNAlater (right) and extracted using the QIAamp PowerFecal Pro DNA Kit. Created with BioRender.com.

When comparing DNA yield across all methods for the three initially-tested avian species (chicken, ostrich, and kākāpō), the Perry-West method returned the highest average yield for both chicken and ostrich faeces (Figure 2A), whilst the MicroGEM PrepGEM Bacteria Kit returned the highest average yield for kākāpō (Figure 2A). Choice of preservation method impacted extraction kit performance, with faecal samples stored in RNAlater returning a higher yield than those stored in 95% ethanol regardless of extraction method. Samples stored in RNAlater also gave better A260/A280 nm values overall (Figure 2B). For chicken samples, the IndiSpin Pathogen Kit, PowerFecal Pro DNA Kit, and the ZymoBIOMICS Miniprep Kit most consistently returned A260/A280 nm values of ~1.8, whilst for ostrich this was only the case for the PowerFecal Pro and ZymoBIOMICS Miniprep kits. For kākāpō, the QIAamp PowerFecal Pro DNA Kit and the ZymoBIOMICS Miniprep Kit returned values of ~1.8 when samples were stored in RNAlater, but the IndiSpin Pathogen Kit returned a value of 1.8 when samples were stored in 95% ethanol (Figure 2B). With 95% ethanol as the preservation method, only the IndiSpin Pathogen Kit was reproducible (coefficient of variation (CV) below 1) for both DNA yield and DNA purity (CV_yield_ = 0.11, CV_purity_ = 0.78). When the method of preservation was RNAlater, three kits were reproducible for both yield and purity: IndiSpin Pathogen Kit (CV_yield_ = 0.85, CV_purity_ = 0.57), QIAamp PowerFecal Pro DNA Kit (CV_yield_ = 0.89, CV_purity_ = 0.16), and the ZymoBIOMICS DNA Miniprep Kit (CV_yield_ = 0.74, CV_purity_ = 0.10) (Supplementary Table S1).

Differences between preservation in 95% ethanol versus RNAlater were particularly evident when visualising extracted DNA on an agarose gel (Figure 2C; Supplementary Figures S3–S7). DNA integrity, exemplified by a single dense band of DNA as opposed to a smear, was in virtually all cases much better for the samples preserved in RNAlater.

#### 3.1.2. 16S rRNA gene-based microbiota profiles

**Figure 3:**
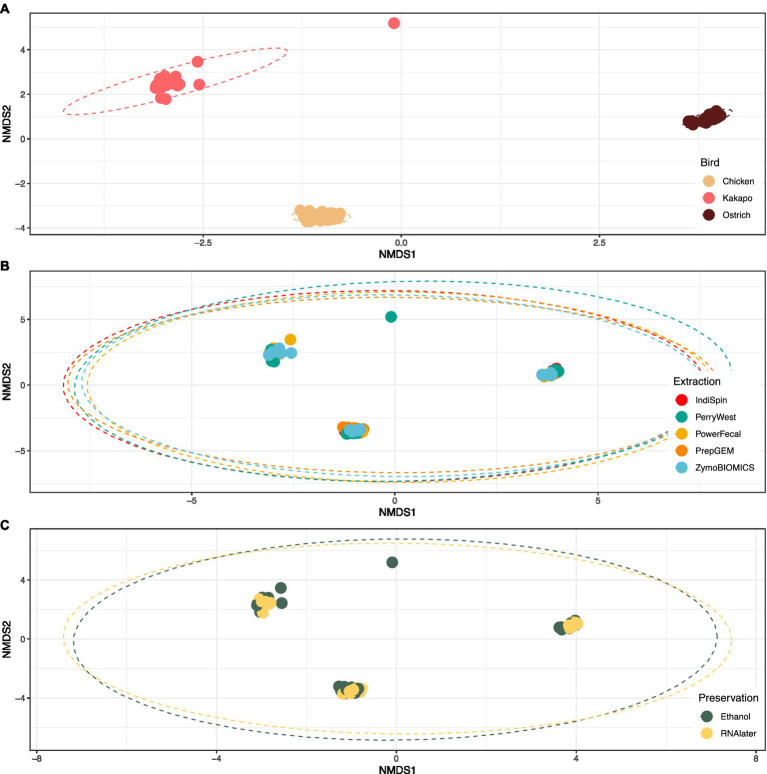
Influence of avian species, DNA extraction method and preservation method on 16S rRNA gene-based microbiota profiles. Non-metric multidimensional scaling (nMDS) plot of avian gut microbiota partitioned by **(A)** avian species, **(B)** extraction method, and **(C)** preservation method. The plot shows strong clustering by avian species, and no clustering by extraction or preservation method. Stress = 0.05 for all plots.

Avian species explained 69.8% of variation in bacterial community profiles (PERMANOVA; *F* = 113.7, *p* < 0.001), compared to just 4.7% of variation explained by DNA extraction method (*F* = 3.80, *p* < 0.001) and 1.2% explained by preservation method (*F* = 3.96, *p* < 0.01). The bacterial communities of chicken, kākāpō and ostrich samples clustered strongly by avian species, irrespective of either preservation or extraction method (Figure 3).

**Figure 4:**
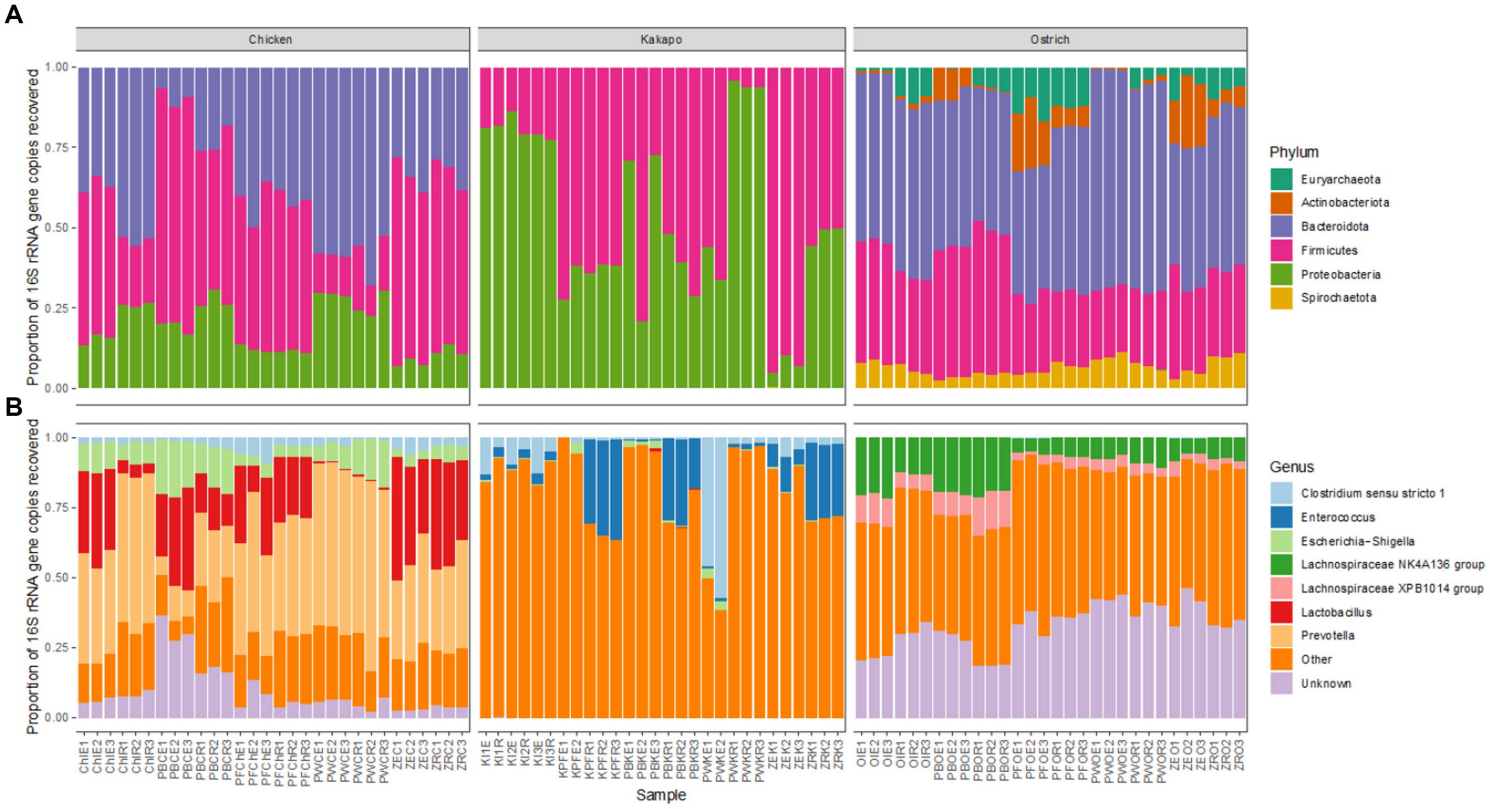
Influence of preservation and DNA extraction method on taxonomic distribution of bacteria within avian faeces. Relative 16S rRNA gene sequence abundance of the most abundant (>1%) bacterial **(A)** phyla and **(B)** genera within chicken, kākāpō, and ostrich samples (*n* = 30 per host species).

The 16S rRNA gene based taxonomic profiles of each avian species showed some variation amongst different combinations of preservation and extraction method at both bacterial phylum and genus level, but much more pronounced differences amongst host avian species (Figure 4). The overriding influence of host species was consistent with the aforementioned nMDS (Figure 3) and PERMANOVA findings. Chicken and ostrich samples showed high levels of the phyla *Bacteroidota* and *Firmicutes*, whilst kākāpō contained mostly *Gammaproteobacteria* and *Firmicutes*. Ostriches also contained members of the archaeal phylum *Euryarchaeota* (Figure 4). It is worth noting that whilst the dominant phyla and genera were broadly consistent for a given host species across preservation and extraction methods, the relative proportions of some taxa did vary substantially amongst replicates.

### 3.2. Applying selected DNA preservation and extraction approach to the analysis of diverse avian species

Following evaluation of DNA quality and yield, DNA integrity was visualised with gel electrophoresis (Figure 2; Supplementary Figure S1), and 16S rRNA gene amplicons were sequenced (Figures 3, 4). From these results, we chose a combination of preservation in RNAlater followed by DNA extraction with the QIAamp PowerFecal Pro DNA Kit for testing on a further 10 ecologically and taxonomically diverse avian species. We freely acknowledge that whilst RNAlater was clearly better in our hands compared with 95% ethanol, several of the tested DNA extraction approaches performed equally or nearly as well as the QIAamp PowerFecal kit. Ultimately we chose the latter due to its consistency in returning DNA purity A260/A280 nm values close to the desired 1.8 ratio, its relatively high DNA yield, and its high DNA integrity on the agarose gel, as well as ease of use.

#### 3.2.1. DNA quality, quantity and integrity

**Figure 5:**
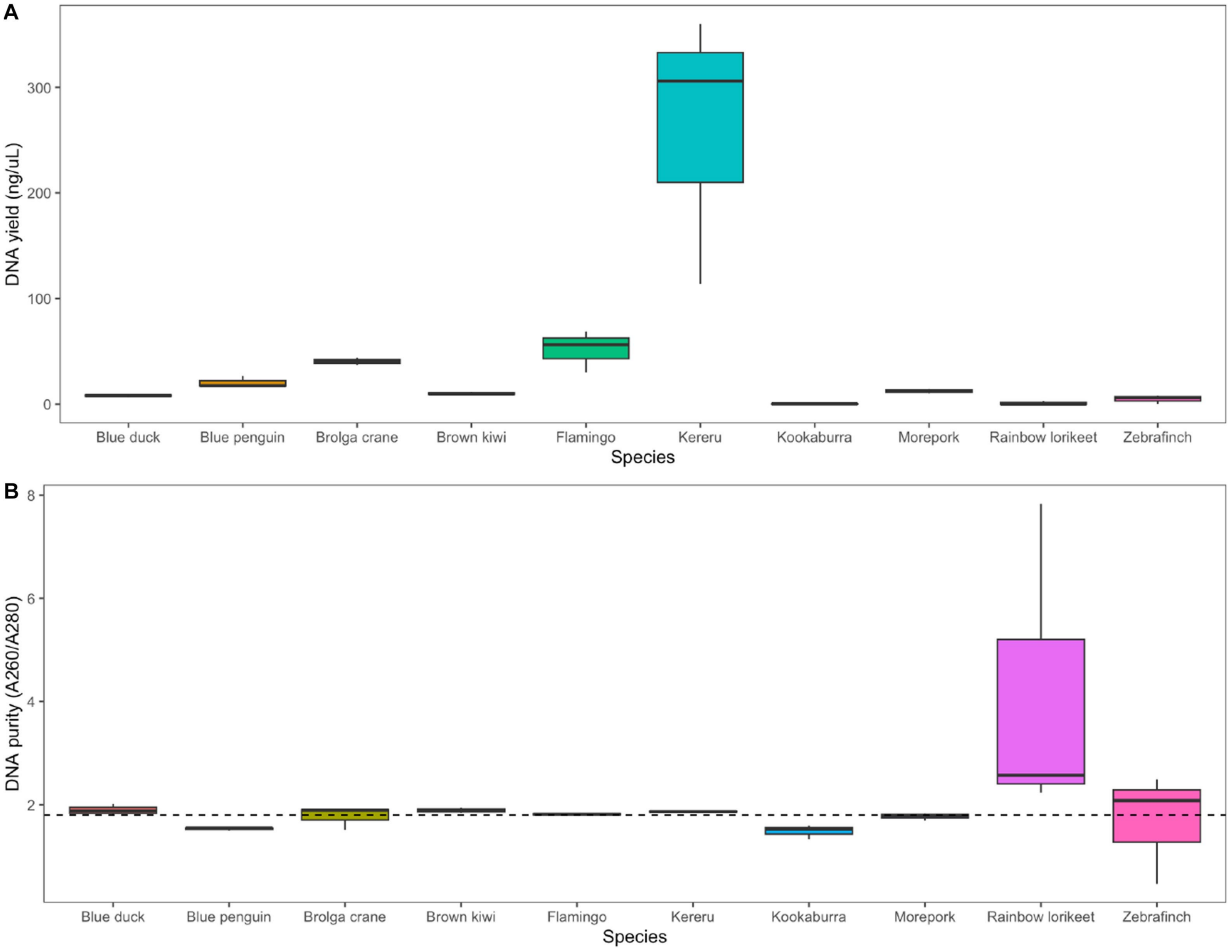
Application of the selected preservation/extraction methods combination to faecal samples from 10 avian species. **(A)** DNA yield (ng/μL) and **(B)** DNA purity (A260/A280 nm ratio) of faecal samples from 10 avian species preserved in RNAlater and extracted using the QIAamp PowerFecal Pro DNA Kit (*n* = 3 technical replicates per host species). The dotted line in panel **(B)** represents the 1.8 nm ratio, the desired quality for DNA in downstream analysis. Agarose gel images showing extracted DNA for all avian species are included in the Supplementary material.

We extracted measurable quantities of DNA from all avian species using the chosen combination of RNAlater preservation and QIAamp PowerFecal Pro DNA Kit extraction, with the highest yields obtained for kereru and flamingo (Figure 5A). The A260/A280 nm ratio was close to the desired 1.8 value for all species except rainbow lorikeet (Figure 5B). Agarose gel electrophoresis allowed us to visualise extracted DNA for most, but not all, of the analysed avian species (Supplementary Figure S8).

#### 3.2.2. 16S rRNA gene-based microbiota profiles

**Figure 6:**
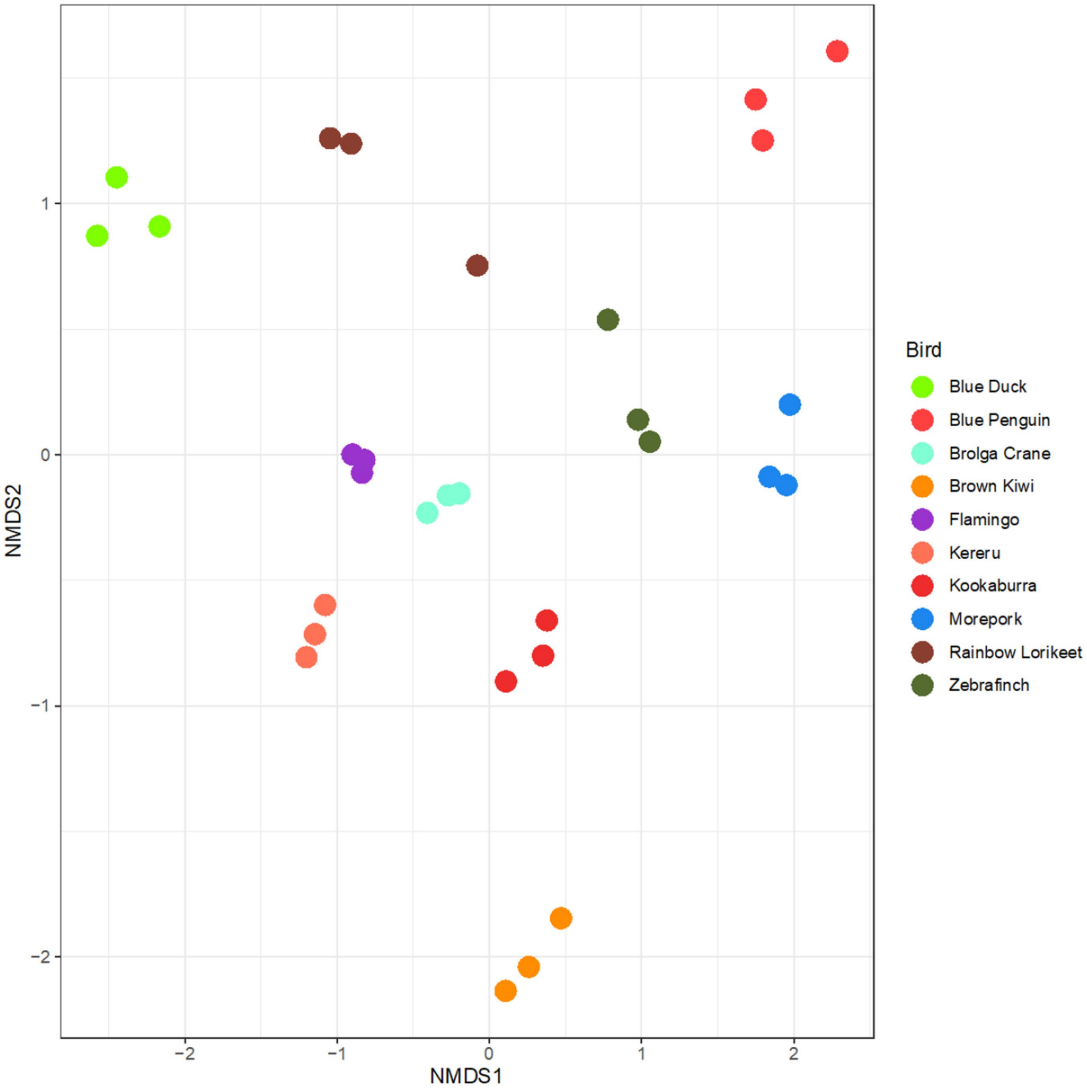
Microbiota profiles of faecal samples from 10 avian species obtained using the selected preservation/extraction methods combination. nMDS plot represents 16S rRNA gene-based microbiota profiles for all avian samples preserved in RNAlater and processed through the QIAamp PowerFecal Pro DNA Kit. The plot largely shows clustering by technical replicate for each avian species. Stress = 0.15.

Upon preserving and extracting faecal samples using the selected uniform methodology, the technical replicates representing bacterial community composition clustered clearly by host avian species (Figure 6). The rainbow lorikeet replicates were the most variable of all analysed birds (Figure 6).

**Figure 7:**
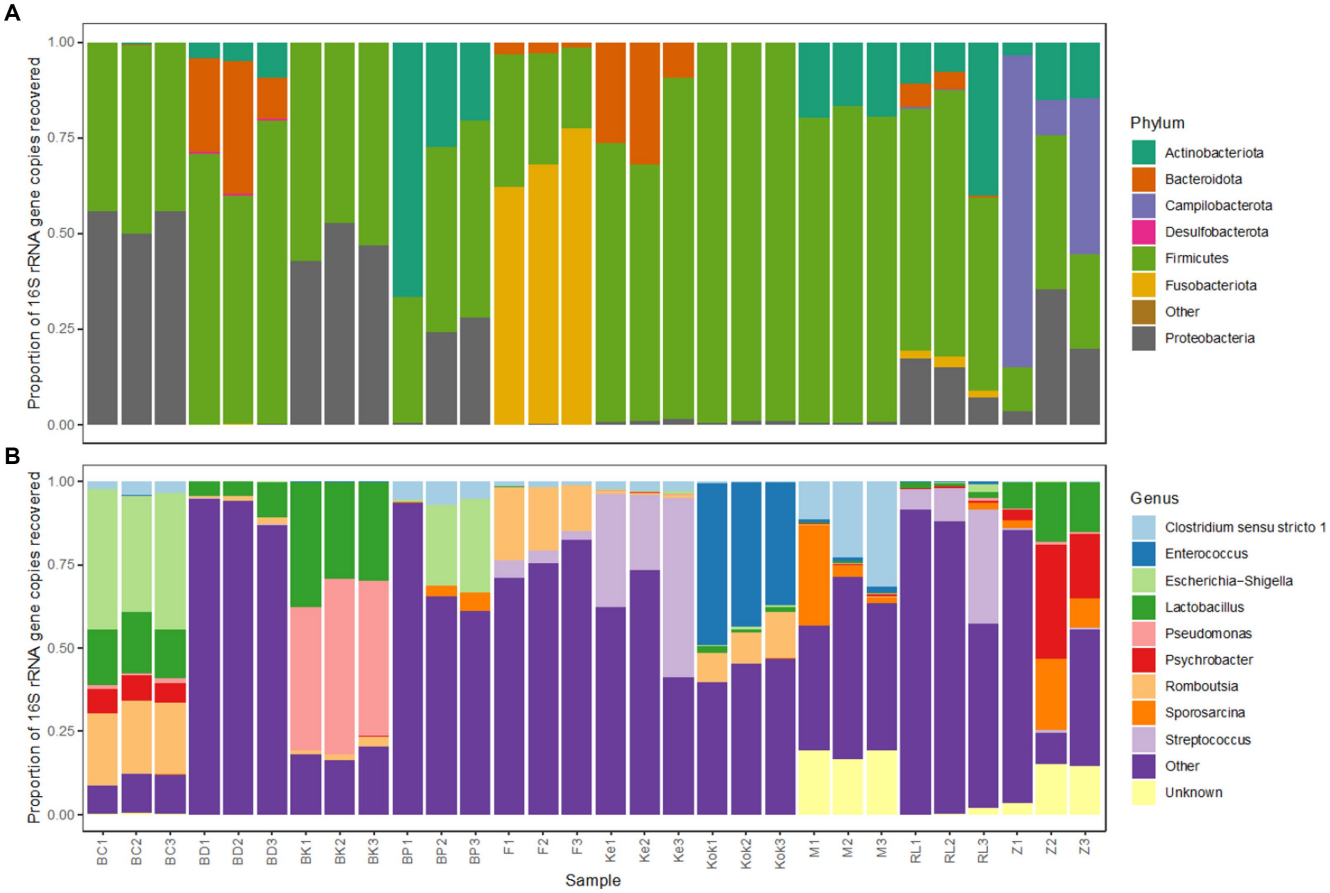
Bacterial taxonomic profiles of faecal samples from 10 avian species obtained using the selected preservation/extraction combination. Relative 16S rRNA gene sequence abundance for the most abundant (>1%) bacterial **(A)** phyla and **(B)** genera found in faecal samples obtained from 10 birds (brolga crane, BC; blue duck, BD; brown kiwi, BK; blue penguin, BP; flamingo, F; kereru, Ke; kookaburra, Kok; morepork, M; rainbow lorikeet, RL; zebra finch, Z). Each avian species is represented by 3 technical replicates.

The microbiota profiles for each technical replicate of the further 10 birds analysed strongly resembled one another at both phylum and genus level (Figure 7), as was previously observed for the initial three species. Most birds harboured high levels of the bacterial phyla *Firmicutes* and *Gammaproteobacteria*, though there were some exceptions.

## 4. Discussion

Over the course of our comparisons amongst two preservation methods and five DNA extraction approaches we found variable outcomes for DNA yield, purity, and integrity. Though the quality and quantity of DNA recovered varied considerably amongst the tested combinations of preservation and extraction methods, this ultimately had negligible effect on the recovered bacterial community profiles, which grouped much more strongly by avian species than preservation method or extraction approach. When a chosen combination of methods (RNAlater and QIAGEN PowerFecal) was applied to a further 10 avian species the resulting DNA yield and purity did vary by avian species, but was within an acceptable range for most. These results demonstrate that although DNA of high quality and quantity may be desired for downstream applications, it ultimately appears to have had little impact on the observed 16S rRNA gene-based microbiota profiles.

### 4.1. Studying the avian faecal microbiota: choice of preservation and extraction method strongly influences DNA quantity and quality but not 16S profile

When conducting research on microbial communities, investigators must select from a variety of preservation and extraction methods which will, ideally, deliver DNA of high quality and quantity. These choices are often made on the basis of cost, ease of use, or efficacy, and our tested DNA extraction methods spanned the gamut of these factors. Our findings relating to the two tested preservation methods, 95% ethanol and RNAlater, were quite clear-cut. In addition to the potential to preserve RNA as well as DNA for future analyses, RNAlater performed better in nearly all cases for returning high-quality DNA of any quantity (Figure 2B). This is in contrast to a previous study in which testing of five preservation methods (including RNAlater) on avian faecal samples revealed no significant differences in DNA quantity or purity (Vargas-Pellicer et al., 2019). It is worth noting, however, that the earlier study included only a single bird species and one extraction method. By contrast to our findings for preservation methods, the performance of DNA extraction methods was far less skewed toward one method over any other. Multiple methods returned DNA of high yield when preserved in RNAlater, particularly the QIAGEN PowerFecal kit, MicroGEM prepGEM kit, ZymoBIOMICS DNA kit, and the Perry-West method (Figure 2A). Despite ease of use and speed of extraction, the MicroGEM prepGEM kit provides output DNA for specific purposes and may not be suitable in some contexts. Similarly, whilst the Perry-West method returned some of the highest yields for DNA, it is a lengthy and unwieldy method that is potentially more prone to contamination, and in some cases DNA yield was gained at the expense of DNA integrity. Ultimately, the QIAGEN PowerFecal Pro DNA kit and ZymoBIOMICS DNA Miniprep kit performed similarly well across all three initial avian species, providing a viable option for the extraction of microbial DNA from avian faeces.

Despite the clear variation in performance of the tested preservation and extraction methods, the resulting bacterial community profiles showed remarkably little effect of either preservation or extraction method (Figure 3). This result was striking, as research conducted across multiple study systems and with different extraction methods often returns a strong effect of the applied methodology on observed microbiota profiles (Kennedy et al., 2014; Waite and Taylor, 2014; Vargas-Pellicer et al., 2019; Broderick et al., 2021). Moreover, in our hands several of the tested method combinations returned almost no DNA, or DNA of very low purity. Though much time and attention are devoted to obtaining DNA of high quality and sufficient quantity, it ultimately had an insubstantial influence on observed bacterial community profiles. It should be noted that whilst the dominant bacterial taxa were consistent across method combinations, their relative abundances did differ substantially between some methods, consistent with previous findings (Vargas-Pellicer et al., 2019). For example, the Perry-West method returned higher proportions of some specific bacterial phyla and genera compared to other methods for kākāpō and chicken, in one replicate to the complete exclusion of other taxa (Figure 4). Though PERMANOVA and nMDS results confirmed that neither preservation nor extraction method were strong drivers of observed bacterial community profiles, this shift in relative abundance may reflect the propensity of some methods to select for certain taxa over others which may be more difficult to extract.

### 4.2. A combination of RNAlater and PowerFecal pro is effective across diverse avian species

The extraction of microbial DNA from avian faeces can be particularly difficult, likely due to avian anatomy (Eriksson et al., 2017). Some faeces may be harder to extract than others due to their physico-chemical composition, such as herbivores producing particularly fibrous faecal output. When our chosen method combination was applied to 10 avian species from across the avian phylogeny, the results were largely successful for both DNA yield and desired purity (Figure 5), though faeces obtained from the rainbow lorikeet yielded little DNA and an excessively high A260/A280 nm ratio. Technical replicates returned largely consistent bacterial community profiles (Figure 6), with the major phyla and genera reflecting those commonly reported for vertebrate gut communities.

### 4.3. Limitations

Whilst we believe that this study represents a thorough investigation of DNA preservation and extraction methods for the avian microbiota, we nonetheless acknowledge some limitations. For example, technical replicates based on pooled samples from multiple individuals were used in order to minimise inter-individual variation. This facilitated our evaluation of method-specific differences, but at the same time prevented us from assessing the extent of inter-individual variation, which can be considerable. The inclusion of a mock community, comprising known organisms in known quantities, would also help elucidate how accurately any of the tested approaches reproduce the source bacterial community.

As a further methodological constraint, all birds sampled from this study were in captivity, either from a private residence or at Auckland Zoo. Captivity can influence gut microbiota composition (Clayton et al., 2016; McKenzie et al., 2017; Frankel et al., 2019; San Juan et al., 2021), thus the microbiota found here may be different from those identified in a wild animal. Moreover, the diet of these birds in captivity will differ from that encountered by their wild counterparts, potentially altering faecal compositions and rendering it more or less challenging to extract DNA.

The use of 16S rRNA gene amplicon sequencing is highly effective for determining microbial taxonomy, as we have done in this study, but cannot necessarily be extrapolated to the results obtained from shotgun metagenomics or other sequencing approaches. A further downstream application for many researchers will be functional analysis of the microbiota, which may require higher quantities of DNA and/or higher molecular weight DNA for long-read sequencing as enabled by PacBio or Nanopore (Mayjonade et al., 2016; Trigodet et al., 2022). Although all method combinations returned sufficient DNA for 16S rRNA gene sequencing of the bacterial community, further research will be required to determine an optimal approach for extracting DNA for shotgun metagenomics and other sequencing approaches. Extension of the current study to other components of the avian microbiota, such as fungi, also warrants future research attention.

## 5. Concluding remarks

Here we investigated how choice of DNA preservation and extraction method influences avian bacterial community profiles. Whilst the quality and quantity of recovered DNA varied considerably amongst the tested combinations of preservation and extraction methods, this – surprisingly – had negligible effect on the recovered 16S rRNA gene-based bacterial community profiles. We ultimately chose a combination of preservation with RNAlater and extraction with the QIAamp PowerFecal Pro DNA Kit, but acknowledge that several of the tested extraction approaches performed near or equally well and should be eminently suitable for future studies of the avian gut microbiota.

## Data availability statement

The datasets presented in this study can be found in online repositories. The names of the repository/repositories and accession number(s) can be found at: https://www.ncbi.nlm.nih.gov/genbank/, PRJNA981578.

## Ethics statement

Ethical review and approval was not required for the animal study because Faecal samples from birds were collected opportunistically at Auckland Zoo. At no point were birds handled or disturbed during faecal sample collection.

## Author contributions

MT: study conceptualisation. JE and AP: sample collection. JE: experimental work. JE, CH, and AW: bioinformatics. JE, CH, AW, AP, and MT: manuscript writing and editing. All authors contributed to the article and approved the submitted version.

## Funding

CH gratefully acknowledges the support of Fulbright New Zealand.

## Conflict of interest

AP is employed by Auckland Zoo.

The remaining authors declare that the research was conducted in the absence of any commercial or financial relationships that could be construed as a potential conflict of interest.

## Publisher’s note

All claims expressed in this article are solely those of the authors and do not necessarily represent those of their affiliated organizations, or those of the publisher, the editors and the reviewers. Any product that may be evaluated in this article, or claim that may be made by its manufacturer, is not guaranteed or endorsed by the publisher.
