# Supplementary material for: 16S rRNA gene-based microbiota profiles from diverse avian faeces are largely independent of DNA preservation and extraction method
Source: Front Microbiol. 2023 Aug 22;14:1239167. doi: 10.3389/fmicb.2023.1239167 (PMC10477782; doi:10.3389/fmicb.2023.1239167)
Supplement: Supplementary file 1 [file Data_Sheet_1.pdf]

Supplemental material for:

16S rRNA gene-based microbiota profiles from diverse avian faeces are largely independent of DNA preservation and extraction method

Johnson Edwards, Carmen Hoffbeck, Annie G. West, An Pas, Michael W. Taylor

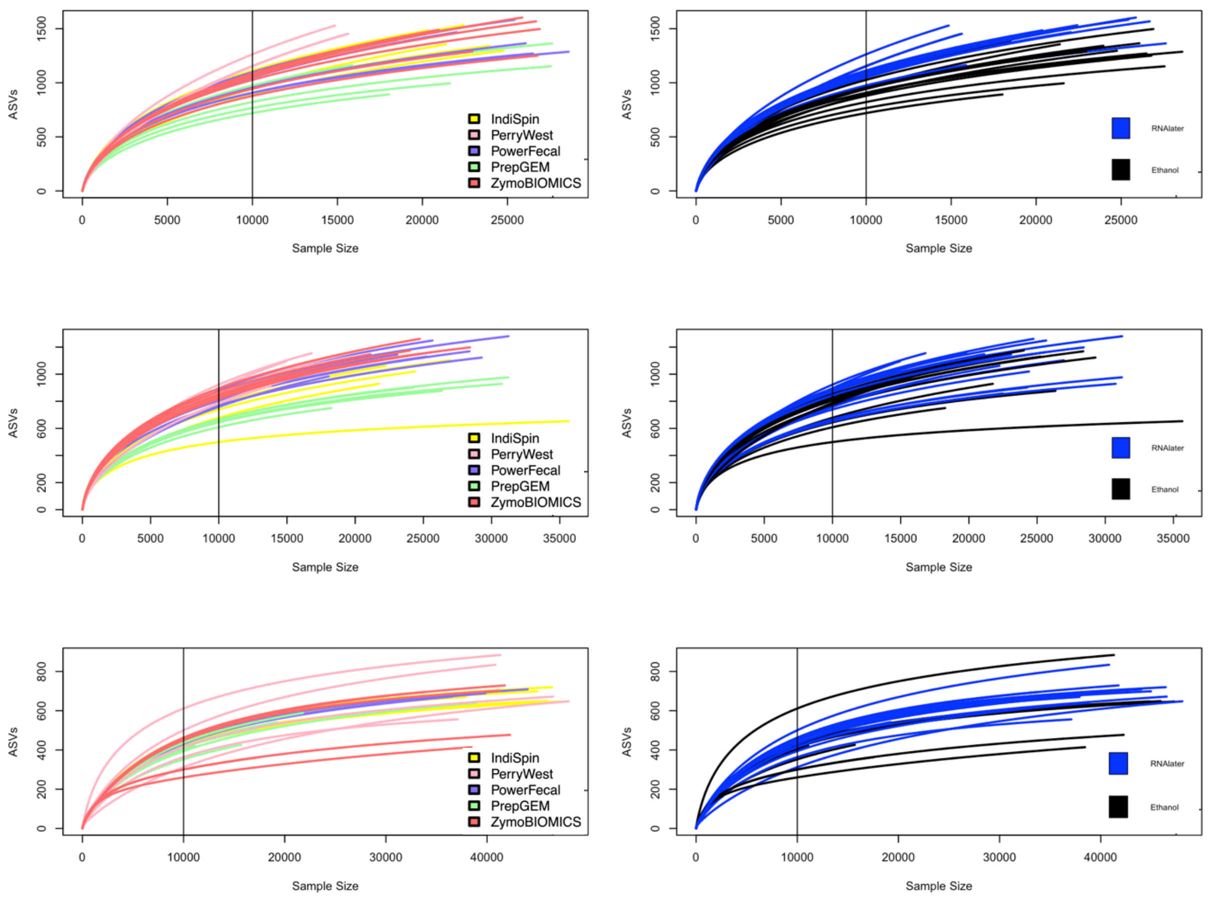

Figure S1. Rarefaction plots showing the number of ASVs recovered from faecal samples of (A) chicken, (B) ostrich, and (C) kākāpō grouped by extraction method (left) and preservation method (right).

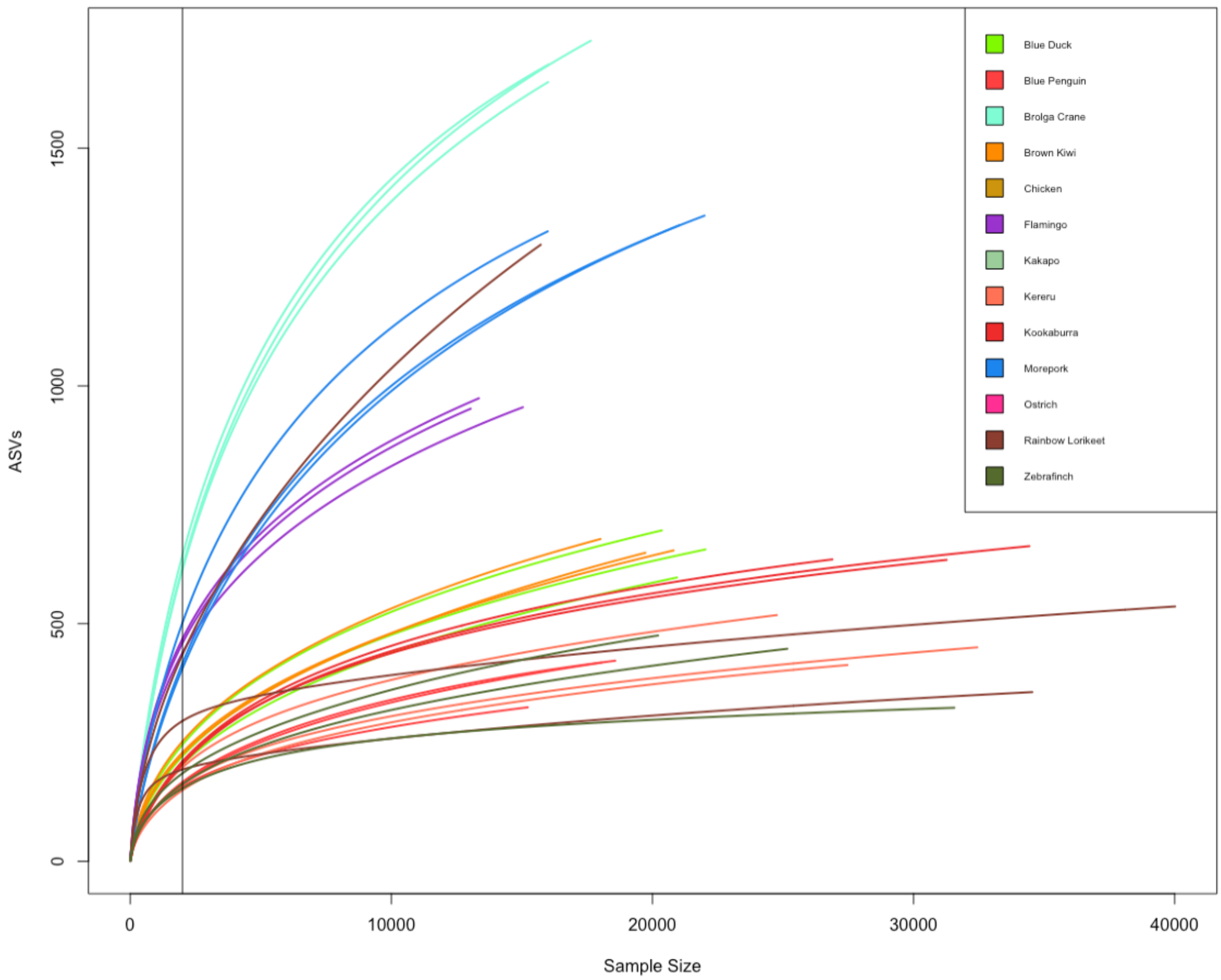

Figure S2. Rarefaction plot showing the number of ASVs recovered from faecal samples of 13 bird species preserved in RNAlater and extracted with the QIAamp PowerFecal Pro DNA Kit. The black vertical line shows the depth to which all samples were rarefied.

Table S1. Coefficient of variation (CV) values indicating the reproducibility of each DNA extraction method for DNA yield and purity with either 95% ethanol or RNAlater as the preservation method. Values less than 1.0 are in bold type.

		IndiSpin Pathogen Kit	QIAamp PowerFecal Pro DNA Kit	MicroGEM PrepGEM Bacteria Kit	Perry-West method	ZymoBIOMICS DNA miniprep Kit
95% ethanol	Yield	**0.78**	1.50	1.23	1.50	1.51
	Purity	**0.11**	**0.44**		**0.25**	**0.96**
RNAlater	Yield	**0.85**	**0.89**	1.00	1.32	**0.74**
	Purity	**0.57**	**0.16**		**0.15**	**0.10**

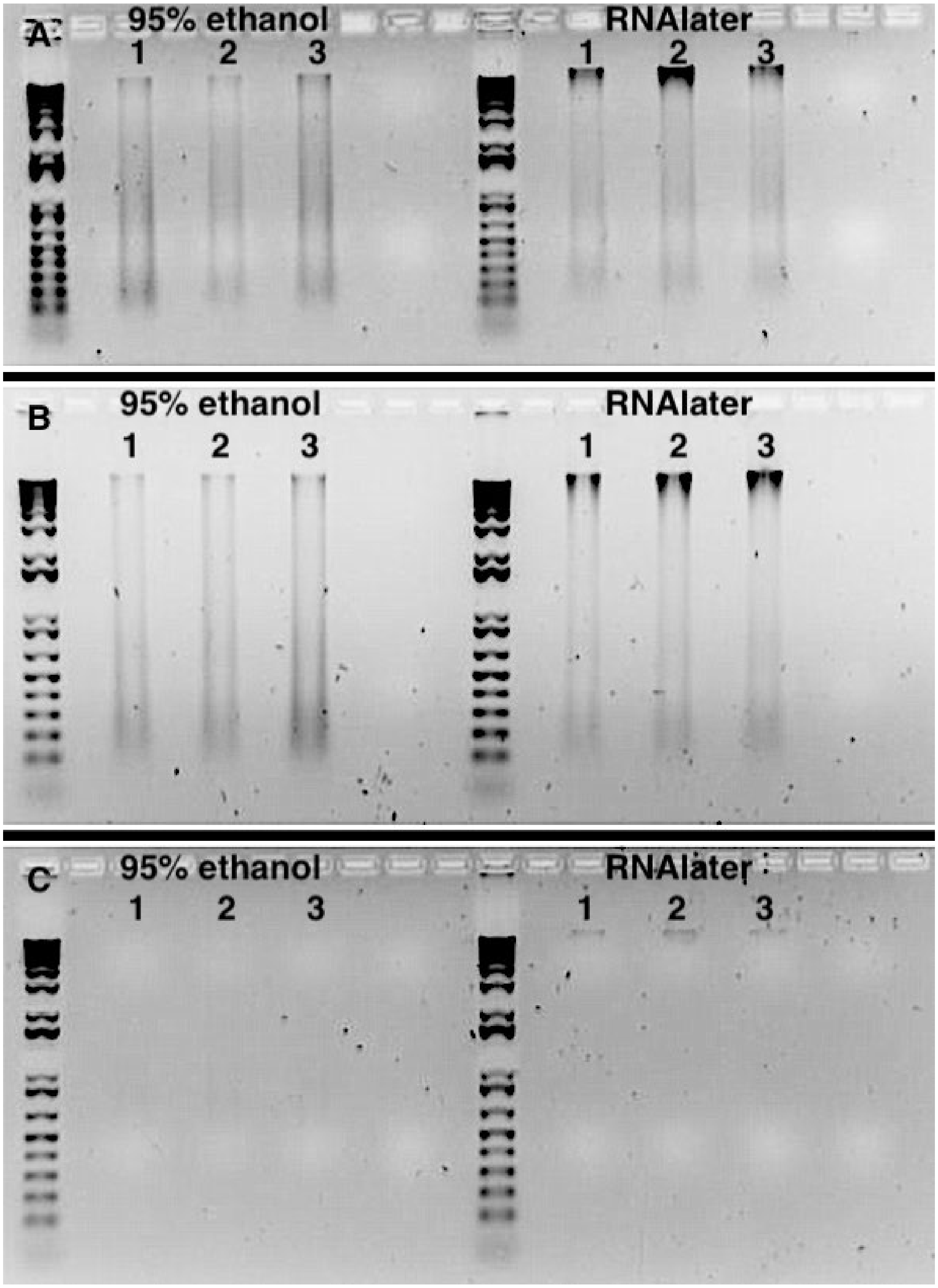

Figure S3: Integrity of DNA extracted using the IndiSpin Pathogen Kit for (A) chicken, (B) ostrich, and (C) kākāpō. Samples stored in 95% ethanol showed more shearing in all cases than samples stored in RNAlater, and even those in RNAlater were barely visible for kākāpō samples. Numbers 1-3 represent technical replicates from each species.

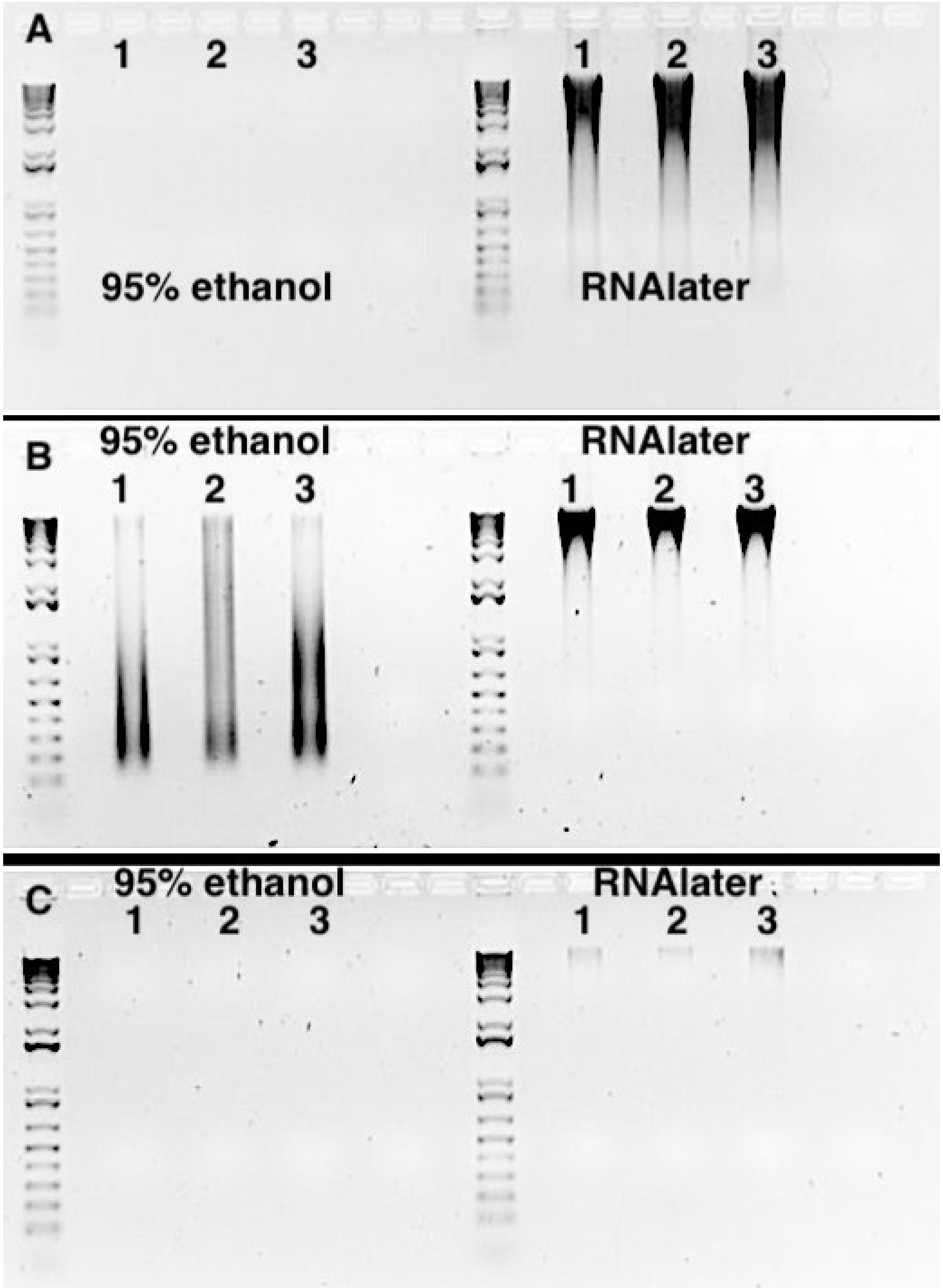

Figure S4: Integrity of DNA extracted using the QIAGEN PowerFecal Pro DNA Kit for (A) chicken, (B) ostrich, and (C) kākāpō. Samples stored in 95% ethanol showed more shearing in all cases than samples stored in RNAlater, and even those in RNAlater were barely visible for kākāpō samples. Numbers 1-3 represent technical replicates from each species.

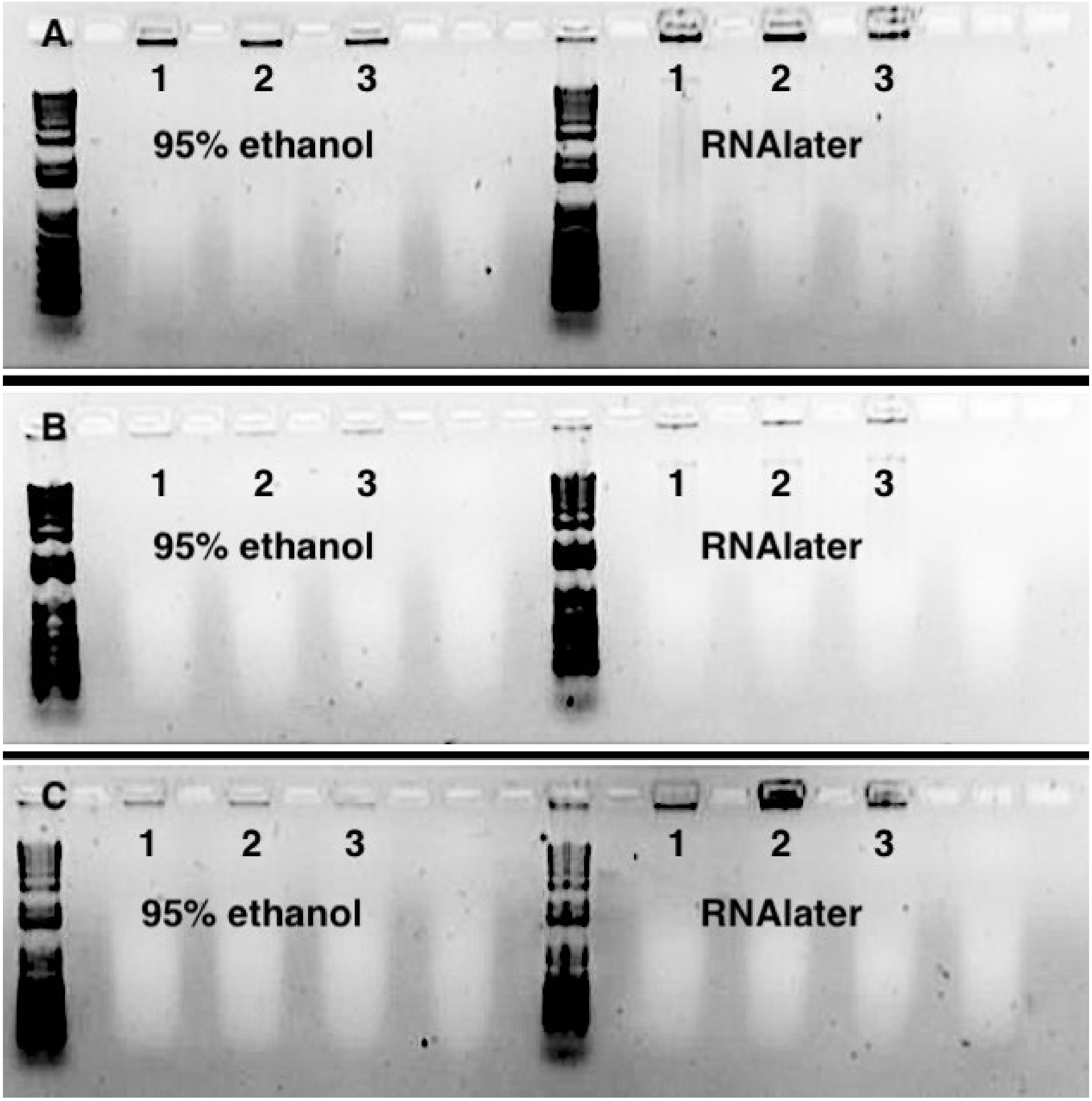

Figure S5: Integrity of DNA extracted using the MicroGEM PrepGEM bacteria kit for (A) chicken, (B) ostrich, and (C) kākāpō. Samples stored in 95% ethanol showed more shearing in all cases than samples stored in RNAlater, and even those in RNAlater were barely visible. Numbers 1-3 represent technical replicates from each species. Note that the bands at the top of the gel are not nucleic acids, but enzymes and proteins formed during the lysis reaction.

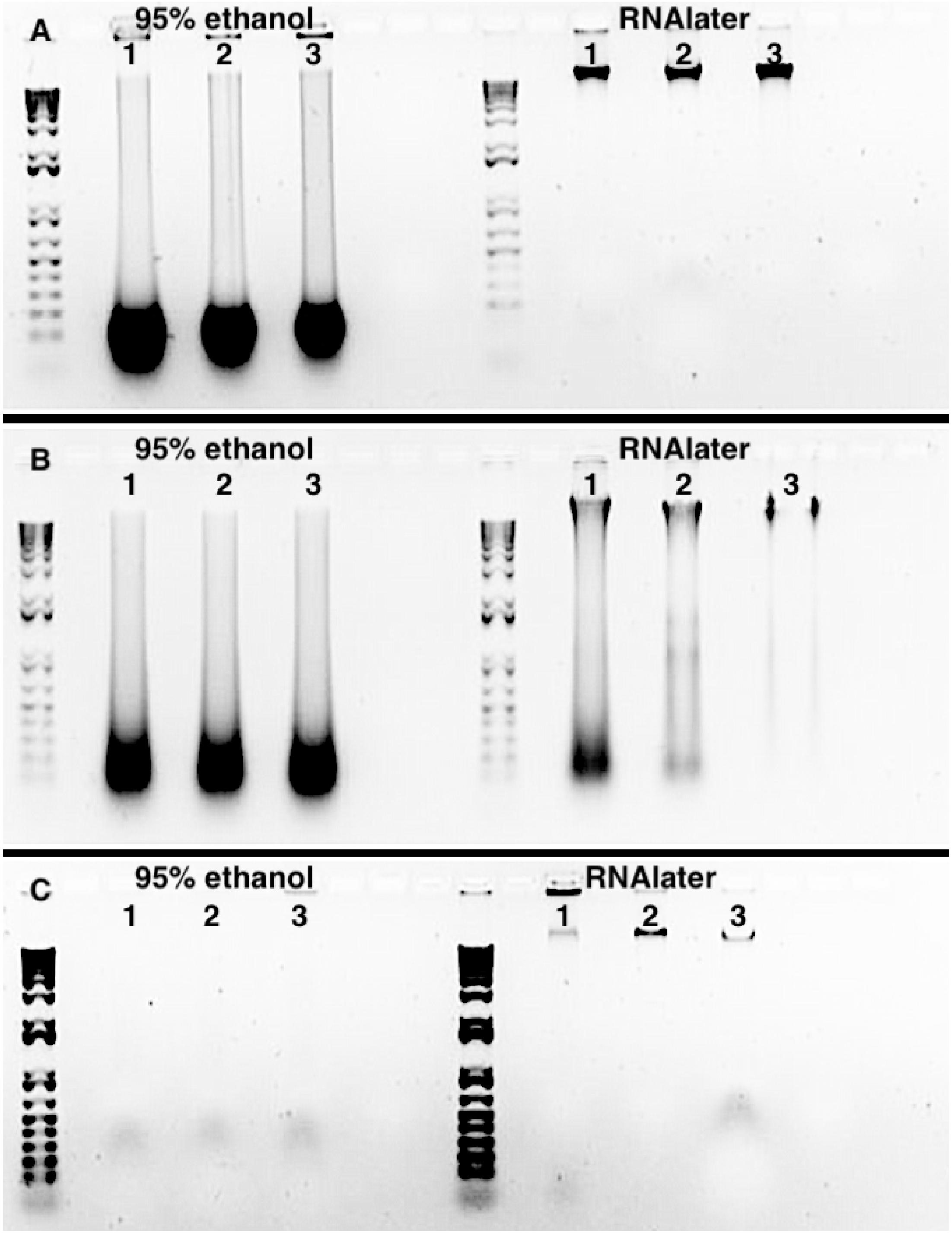

Figure S6: Integrity of DNA extracted using the Perry-West method for (A) chicken, (B) ostrich, and (C) kākāpō. Samples stored in 95% ethanol showed more shearing in all cases than samples stored in RNAlater, and even those in RNAlater were barely visible for chicken and kākāpō. Numbers 1-3 represent technical replicates from each species.

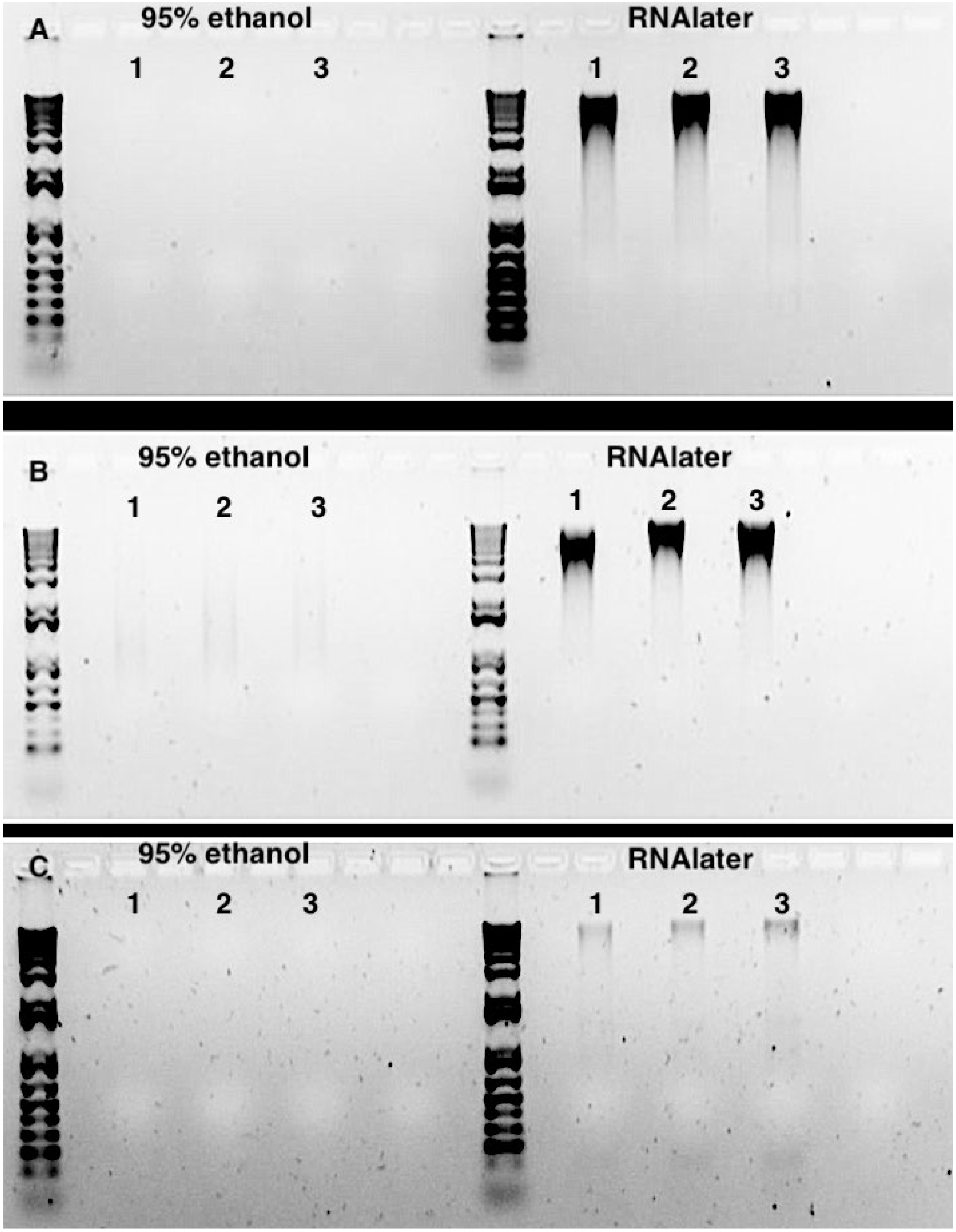

Figure S7: Integrity of DNA extracted using the ZymoBIOMICS DNA Miniprep Kit for (A) chicken, (B) ostrich, and (C) kākāpō. Samples stored in 95% ethanol showed more shearing in all cases than samples stored in RNAlater, and even those in RNAlater were barely visible for kākāpō. Numbers 1-3 represent technical replicates from each species.

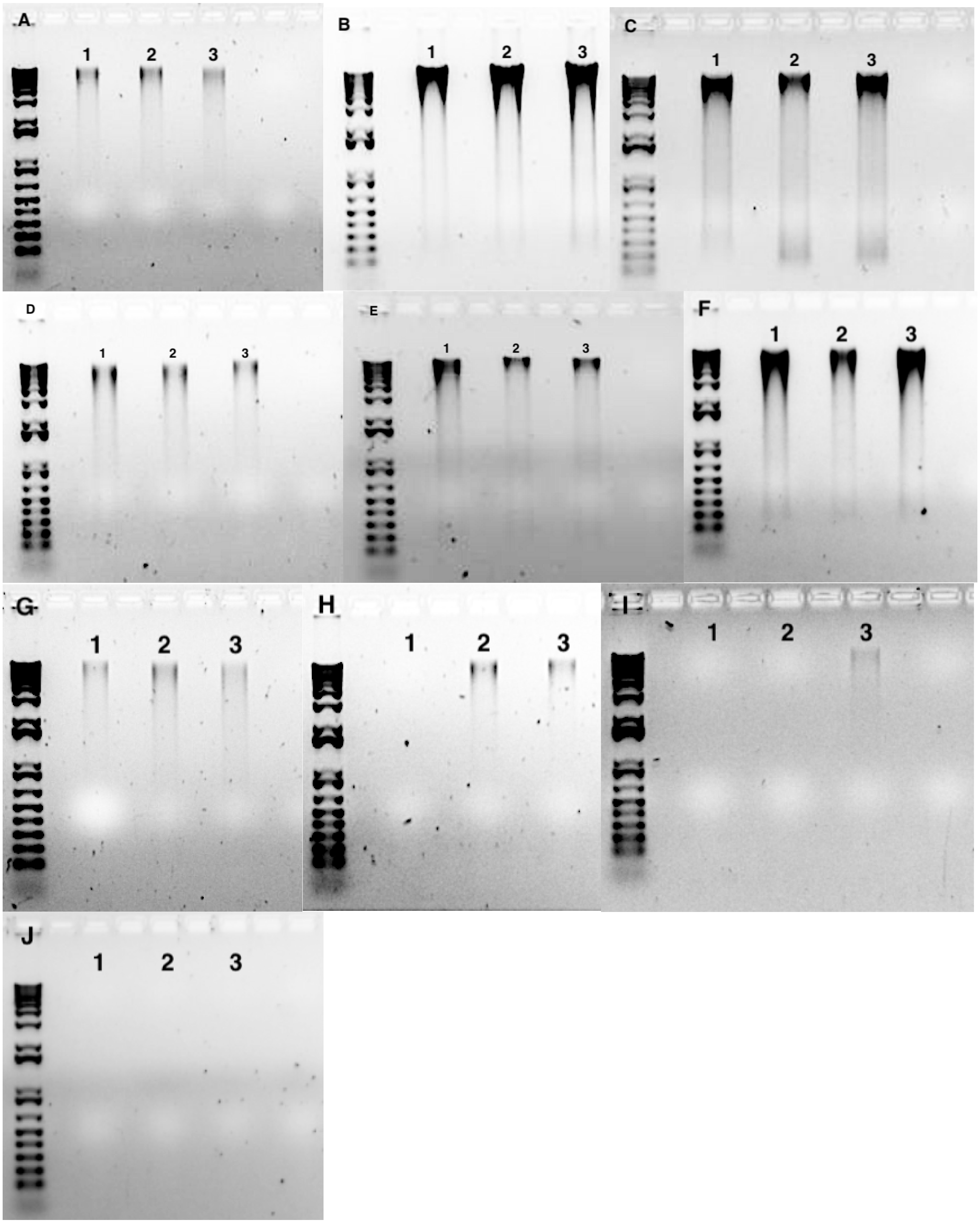

Figure S8: Integrity of DNA extracted using PowerFecal Pro DNA kit in RNAlater from (A) brown kiwi, (B) kereru, (C) brolga crane, (D) morepork, (E) blue penguin, (F) flamingo, (G) blue duck, (H) zebrafinch, (I) rainbow lorikeet, and (J) kookaburra. Numbers 1-3 represent technical replicates from each species.
